# Analysis of the incidence status and risk factors for immune-mediated neuropathies: a single-center case–control study

**DOI:** 10.3389/fneur.2026.1733557

**Published:** 2026-02-09

**Authors:** Yunlong Wang, Zhanchang Feng, Lingxiu Liu, Mengchao Wan

**Affiliations:** 1 Department of Oncology, Jiangxi Provincial People's Hospital, The First Affiliated Hospital of Nanchang Medical College, Nanchang, China; 2 Department of Outpatient, Jiangxi Provincial People's Hospital, The First Affiliated Hospital of Nanchang Medical College, Nanchang, China

**Keywords:** Guillain-Barré syndrome, immune-mediated neuropathies, incidence, logistic regression analysis, preceding infection, risk factors

## Abstract

**Objective:**

To investigate the epidemiological characteristics of immune-mediated neuropathies (primarily including Guillain-Barré Syndrome and Chronic Inflammatory Demyelinating Polyradiculoneuropathy) and systematically analyze their associated risk factors, providing a theoretical basis for disease prevention and early intervention.

**Methods:**

A retrospective study was conducted. 158 patients with immune-mediated neuropathies admitted to our hospital between January 2020 and December 2023 were selected as the case group, while 160 healthy individuals undergoing physical examinations during the same period were selected as the control group. Demographic data and clinical records (including history of preceding infections, vaccination history, history of comorbid diseases, etc.) were collected and compared for all subjects. Univariate analysis and multivariate logistic regression analysis were used to identify independent risk factors for immune-mediated neuropathies.

**Results:**

The annual incidence of immune-mediated neuropathies was 1.24 per 100,000, with a bimodal age distribution (20–30 years and 50–70 years). The incidence was slightly higher in males than in females (1.5:1). Univariate analysis showed statistically significant differences (*p* < 0.05) between the case and control groups in terms of history of preceding infection (particularly respiratory and gastrointestinal infections), recent vaccination history, history of surgical trauma, and comorbid autoimmune diseases. Multivariate logistic regression analysis further confirmed that preceding *Campylobacter jejuni* infection (OR = 4.52, 95% CI: 2.81–7.26, *p* = 0.000), Cytomegalovirus infection (OR = 3.18, 95% CI: 1.95–5.19, *p* = 0.000), recent surgical trauma (OR = 2.45, 95% CI: 1.40–4.29, *p* = 0.002), and the presence of an underlying autoimmune disease (OR = 2.90, 95% CI: 1.72–4.89, *p* = 0.000) were independent risk factors for the onset of immune-mediated neuropathies.

**Conclusion:**

The onset of immune-mediated neuropathies is closely related to various environmental and individual factors. Preceding infections (especially *Campylobacter jejuni* and Cytomegalovirus) are the most significant independent risk factors. In clinical practice, high suspicion for immune-mediated neuropathies should be maintained for individuals with the aforementioned risk factors who present with neurological symptoms, to achieve early diagnosis and treatment.

## 1 Introduction

Immune-mediated neuropathies are a group of acquired disorders caused by an abnormal immune response attacking peripheral nerve structures, leading to demyelination and/or axonal damage (1). Their clinical manifestations are diverse, ranging from acutely onset, rapidly progressive quadriplegia to chronic, protracted symmetric weakness and sensory disturbances. Severe cases can be life-threatening due to respiratory muscle paralysis or result in long-term disability, placing a heavy burden on patients, families, and society (2). Guillain-Barré Syndrome (GBS) and Chronic Inflammatory Demyelinating Polyradiculoneuropathy (CIDP) are the most common and representative conditions within this disease spectrum.

GBS, as a typical acute inflammatory demyelinating polyradiculoneuropathy, has an annual incidence of approximately 0.8–1.9 per 100,000 and is one of the important causes of acute flaccid paralysis (3). CIDP, on the other hand, follows a chronic progressive or relapsing–remitting course, with a prevalence of about 0.8–8.9 per 100,000. If not diagnosed promptly and treated appropriately, it carries a very high disability rate (4). It is widely believed that the pathogenesis of immune-mediated neuropathies is not caused by a single factor, but rather results from a dysregulated immune system in genetically susceptible individuals, triggered by specific environmental factors, leading to cross-immune attacks on peripheral nerve components through mechanisms such as “molecular mimicry” (5, 6). Recent studies continue to support and refine this model, highlighting the complex interplay between host factors and environmental triggers (7).

Numerous studies have shown that preceding infections, particularly with *Campylobacter jejuni*, Cytomegalovirus (CMV), and Epstein–Barr virus (EBV), are the most important antecedent events and risk factors for GBS (8, 9). A systematic review from 2023 reaffirmed the strong association between *C. jejuni* infection and the axonal variant of GBS, particularly in East Asian populations (10). Additionally, vaccination, surgery, trauma, pregnancy, and comorbid autoimmune diseases are also considered potentially associated with disease initiation or relapse (8, 11). However, the incidence and risk factor profiles may vary across different regions and populations, and the interactions between these factors and their exact pathogenic mechanisms still require further elucidation (11).

Given the acute onset, severe condition, and variable prognosis of immune-mediated neuropathies, early identification of high-risk factors is crucial for disease warning, early diagnosis, and intervention (12). Currently, large-sample clinical epidemiological and multifactorial comprehensive analysis studies targeting this disease group are relatively limited in China. Therefore, this study aims to systematically investigate the clinical incidence and characteristics of immune-mediated neuropathies and thoroughly analyze their associated risk factors through a retrospective case–control analysis, to provide valuable clinical epidemiological evidence for identifying high-risk populations, formulating prevention strategies, and improving patient outcomes.

## 2 Materials and methods

### 2.1 Study subjects

A retrospective case–control study design was adopted. Case group: Patients diagnosed with immune-mediated neuropathies and hospitalized in the Department of Neurology of our hospital between January 2020 and December 2023 were selected. Inclusion criteria: ① Meeting the diagnostic criteria for GBS and its subtypes according to the “Chinese Guidelines flor the Diagnosis and Treatment of Guillain-Barré Syndrome 2019,” or meeting the diagnostic criteria for CIDP according to the “Chinese Guidelines for the Diagnosis and Treatment of Chronic Inflammatory Demyelinating Polyradiculoneuropathy 2021” (13); ② Complete clinical data and follow-up information. Exclusion criteria: ① Peripheral neuropathies due to other causes (e.g., diabetic, toxic, metabolic, paraneoplastic, etc.); ② Comorbid severe cardiac, hepatic, renal insufficiency or other major systemic diseases; ③ Missing clinical data. A total of 158 patients were finally included as the case group. Control group: Using 1:1 matching, 160 healthy individuals from the Health Management Center of our hospital during the same period, matched for age and sex with the case group, were randomly selected as the control group. All control subjects had no history or clinical manifestations of neurological diseases. This study was conducted in accordance with the ethical guidelines of the Declaration of Helsinki and was approved by the Ethics Committee of Jiangxi Provincial People’s Hospital (Ethics approval number: No. 2019-05-28). Written informed consent was obtained from all participants prior to the study.

This study was reported in accordance with the Strengthening the Reporting of Observational Studies in Epidemiology (STROBE) guidelines for case–control studies.

### 2.2 Research methods

Data collection: The following information was collected for all subjects by reviewing the electronic medical record system: General demographic data: age, sex. Clinical data: For the case group, the season of onset, clinical symptoms, diagnostic subtype, cerebrospinal fluid examination results, electrophysiological examination results, etc., were collected. Risk factor exposure history: The following situations within 4 weeks prior to onset (case group) or within 4 weeks prior to enrollment (control group) were recorded in detail for both groups: ① History of preceding infection (respiratory infection, gastrointestinal infection, and specific pathogen test results, such as *Campylobacter jejuni* serology, CMV/EBV IgM antibodies); ② Vaccination history (vaccine type and time of administration); ③ History of surgery or trauma; ④ History of comorbid autoimmune diseases (e.g., SLE, rheumatoid arthritis, autoimmune thyroiditis, etc.).

### 2.3 Statistical analysis

Data processing and analysis were performed using SPSS 26.0 statistical software. Measurement data: Normally distributed data are expressed as mean ± standard deviation (x̄ ± s), and comparisons between two groups were conducted using independent samples *t*-test. Count data: Expressed as number (percentage) [*n* (%)], intergroup comparisons were performed using chi-square (χ^2^) test or Fisher’s exact test. Risk factor analysis: First, univariate analysis was performed on potential risk factors. Factors with *p* < 0.1 in the univariate analysis were included in a multivariate logistic regression model (forward LR method) to screen for independent risk factors for the onset of immune-mediated neuropathies, and the odds ratio (OR) and its 95% confidence interval (CI) were calculated. Significance level: *p* < 0.05 was considered statistically significant.

## 3 Results

### 3.1 Basic characteristics of the study subjects

A total of 158 patients with immune-mediated neuropathies (case group) and 160 healthy controls (control group) were included in this study. No statistically significant differences were observed in the distribution of gender (Male: 60.1% vs. 55.0%) and mean age (48.7 ± 16.3 years vs. 47.1 ± 15.9 years) between the two groups (*p* > 0.05), indicating that the groups were well-balanced at baseline. However, comparison of various potential risk factors revealed that the exposure rates in the case group were significantly higher than those in the control group regarding a history of preceding infections, recent surgery/trauma, and comorbid autoimmune diseases. Specific data are detailed in Table 1.

**Table 1:** Comparison of baseline data between immune-mediated neuropathy patients and healthy control group [*n* (%)].

Baseline data	Case group (*n* = 158)	Control group (*n* = 160)	*χ*^2^/t value	*p* value
Sex			1.209	0.272
Male	95 (60.1%)	88 (55.0%)		
Female	63 (39.9%)	72 (45.0%)		
Age (years)	48.7 ± 16.3	47.1 ± 15.9	0.921	0.358
History of preceding infection	112 (70.9%)	45 (28.1%)	58.741	**0.000**
Respiratory infection	68 (43.0%)	32 (20.0%)	19.213	**0.000**
Gastrointestinal infection	59 (37.3%)	18 (11.3%)	29.732	**0.000**
*C. jejuni* infection^†^	41 (25.9%)	8 (5.0%)	26.543	**0.000**
CMV infection^†^	33 (20.9%)	10 (6.3%)	14.923	**0.000**
Recent vaccination history^*^	15 (9.5%)	5 (3.1%)	5.714	**0.017**
Recent surgery/trauma history^*^	28 (17.7%)	11 (6.9%)	8.646	**0.003**
Autoimmune disease^#^	26 (16.5%)	10 (6.3%)	8.453	**0.004**

^†^Positive for specific pathogen testing among preceding infections; *“Recent” refers to within 4 weeks before onset; ^#^Includes systemic lupus erythematosus, rheumatoid arthritis, thyroiditis, etc. *p* value <0.05 indicates statistical significance, shown in bold.

### 3.2 Univariate analysis of risk factors for immune-mediated neuropathies

To further assess the strength of association between each factor and the onset of disease, a univariate analysis was performed. As shown in Table 2, a history of preceding infection, particularly specific infections with *Campylobacter jejuni* (OR = 6.66) and Cytomegalovirus (OR = 3.94), showed a strong association with the development of immune-mediated neuropathies. Furthermore, recent vaccination history, recent surgery/trauma, and a baseline autoimmune disease were also significant risk factors (all *p* < 0.05).

**Table 2:** Univariate analysis of risk factors for immune-mediated neuropathies.

Analysis factor	Case group (*n* = 158)	Control group (*n* = 160)	OR value	95% CI	*p* value
History of preceding infection	112 (70.9%)	45 (28.1%)	6.198	3.869–9.927	**0.000**
*C. jejuni* infection	41 (25.9%)	8 (5.0%)	6.656	3.024–14.651	**0.000**
CMV infection	33 (20.9%)	10 (6.3%)	3.935	1.880–8.237	**0.000**
Recent vaccination history	15 (9.5%)	5 (3.1%)	3.25	1.159–9.112	**0.017**
Recent surgery/trauma history	28 (17.7%)	11 (6.9%)	2.909	1.398–6.051	**0.003**
Autoimmune disease	26 (16.5%)	10 (6.3%)	2.923	1.367–6.250	**0.004**

OR, odds ratio; CI, confidence interval. Univariate analysis used chi-square test.*p* value < 0.05 indicates statistical significance, shown in bold.

### 3.3 Multivariate analysis of independent risk factors for immune-mediated neuropathies

To control for potential confounding effects among the factors and identify independent risk factors, the significant factors from the univariate analysis were incorporated into a multivariate logistic regression model. The results, presented in Table 3, identified four factors as independent risk factors for the onset of immune-mediated neuropathies, ranked from highest to lowest risk strength: preceding *Campylobacter jejuni* infection (OR = 4.52), Cytomegalovirus infection (OR = 3.18), comorbid autoimmune disease (OR = 2.90), and recent surgery/trauma history (OR = 2.45).

**Table 3:** Multivariate logistic regression analysis of risk factors for immune-mediated neuropathies.

Variable	*β* value	Wald *χ*^2^ value	OR value	95% CI	*p* value
*C. jejuni* infection	1.508	28.415	4.52	2.812–7.264	**0.000**
CMV infection	1.157	19.632	3.18	1.948–5.189	**0.000**
Recent surgery/trauma	0.896	9.245	2.45	1.398–4.293	**0.002**
Autoimmune disease	1.065	15.887	2.901	1.722–4.889	**0.000**
Constant	−2.101	65.214	0.122	-	**0.000**

Factors with *p* < 0.1 in the univariate analysis were included in the multivariate logistic regression model (forward LR method). The results show that the above four items are independent risk factors for the onset of immune-mediated neuropathies. *p* value < 0.05 indicates statistical significance, shown in bold.

## 4 Discussion

This study systematically explored the incidence and risk factors of immune-mediated neuropathies through a retrospective case–control analysis. Multivariate logistic regression analysis ultimately identified preceding *Campylobacter jejuni* infection, Cytomegalovirus infection, recent surgery/trauma history, and comorbid autoimmune diseases as four independent risk factors for the onset of immune-mediated neuropathies. These findings are consistent with most previous studies and further deepen our understanding of the pathogenesis of this disease. The aforementioned risk factors can be regarded as “triggering events” for the immune system, with the core mechanism being “molecular mimicry” (6, 14). That is, antigens from pathogens (such as lipooligosaccharides of *Campylobacter jejuni*, certain antigenic epitopes of CMV) or antigens released by damaged tissues share structural similarities with specific components of peripheral nerves (such as gangliosides GM1, GD1a, etc.). While the body produces high-titer antibodies and sensitized lymphocytes against these foreign antigens, it mistakenly attacks its own neural structures, triggering a cross-immune reaction, ultimately leading to demyelination, axonal damage, and even neuronal apoptosis, clinically manifesting as acute or chronic neurological deficits (2, 11). The result showing the highest risk for *Campylobacter jejuni* infection (OR = 4.52) in this study strongly supports its status as the most important trigger factor, a finding echoed in recent global surveillance data (15).

Regarding the incidence, the annual incidence rate found in this study (1.24 per 100,000) is similar to most reports domestically and internationally (3, 4). The predominance of male patients in the case group (60.1%) and the bimodal age distribution (20–30 years and 50–70 years) are noteworthy phenomena deserving further exploration. The higher incidence in males may be related to hormone levels, differences in immune response, and varying exposure opportunities to risk factors. The bimodal age distribution might reflect different susceptibility mechanisms: the younger peak may be associated with a more active immune system and greater exposure to infectious agents (16); while the older peak may be closely related to immune system dysfunction (17), increased comorbidities (18), and decreased neural repair capacity (19). A recent European study also reported a similar male predominance and discussed potential sex-based immunological differences underlying this disparity (20). Furthermore, this study again confirmed the key role of preceding infections, with a high percentage (70.9%) of patients having a clear history of infection before onset, predominantly respiratory and gastrointestinal infections, underscoring the importance of detailed inquiry about antecedent history in clinical practice.

The findings of this study have important clinical implications. First, they reinforce the awareness of identifying high-risk populations. For individuals who have recently experienced *Campylobacter jejuni* enteritis or CMV infection, undergone major surgery or severe trauma, or have other autoimmune diseases, clinicians should maintain a high index of suspicion for immune-mediated neuropathies if symptoms like limb weakness or numbness appear, striving for early diagnosis and intervention to improve outcomes. Second, at the public health level, emphasizing food safety to prevent *Campylobacter jejuni* infection has potential positive significance for reducing the incidence of GBS (21). Additionally, although vaccination was identified as a very low-risk trigger factor (OR = 3.25 in this study, but absolute risk is low), the results support enhanced monitoring of individual responses post-vaccination, while simultaneously emphasizing that the overall benefits of vaccination far outweigh its minimal risks, a balance that has been extensively reviewed in the context of large-scale vaccination campaigns (22).

Although several epidemiological studies on immune-mediated neuropathies have been conducted in China, most have focused on GBS alone or were limited by smaller sample sizes. Our study provides updated and comprehensive risk factor analysis based on a recent cohort (2020–2023), which may reflect current epidemiological trends and healthcare exposures. Furthermore, by including both GBS and CIDP within the spectrum of immune-mediated neuropathies, this study offers a broader perspective on shared and distinct risk factors, contributing to the growing body of regional data needed for tailored prevention and clinical vigilance.

Certainly, this study has several limitations. First, as a single-center retrospective study, there are inevitable selection and information biases. Second, the relatively limited sample size may affect the statistical power for certain rare risk factors (such as specific vaccine types). The etiological diagnosis of some preceding infections relied on serological testing, which may have false positives or negatives. Future larger-sample, multi-center prospective cohort studies are needed to further validate the conclusions of this study and to deeply explore the interactions between genetic susceptibility genes (such as HLA typing) and environmental risk factors, thereby providing a more solid theoretical basis for precise prevention and personalized treatment of the disease.

## Data Availability

The original data presented in the study are included in the article/supplementary material. Further inquiries can be directed to the corresponding author.
